# Supplementary material for: Triple-drug therapy with ivermectin, diethylcarbamazine and albendazole for the acceleration of lymphatic filariasis elimination in Kenya: Programmatic implementation and results of the first impact assessment
Source: PLoS Negl Trop Dis. 2024 Jul 8;18(7):e0011942. doi: 10.1371/journal.pntd.0011942 (PMC11257386; doi:10.1371/journal.pntd.0011942)
Supplement: S1 Table — (DOCX) [file pntd.0011942.s001.docx]

**S1 Table. Sample size determination during the baseline survey conducted in 2018.**

| **Survey Timepoint** | **Survey Area** | **Estimated Population** | **Sample size for random cluster survey of children aged 5-9 years** | **Sample size for random cluster survey of adults*** | **Sample size for purposively selected sites of children aged 5-9 years** | **Sample size for purposively selected sites of adults*** |
| --- | --- | --- | --- | --- | --- | --- |
| Baseline | Lamu | 144,000 | 1380 | 1710 | 200 (40 per site x 5 sites) | 200 (40 per site x 5 sites) |
|  | Jomvu | 140,000 | 1380 | 1710 | 200 (40 per site x 5 sites) | 200 (40 per site x 5 sites) |
| Endline | Lamu | 144,000 | 1380 (average of 46 kids per site) | 3150 (an average of 105 adults per site) | 50 per site | 100 per site |
|  | Jomvu | 140,000 | 1380 (average of 46 kids per site) | 3150 (an average of 105 adults per site) | 50 per site | 100 per site |

*During baseline adults were considered as participants aged 10 years, during endline adults were considered as participants aged 18 years
