# Supplementary material for: Triple-drug therapy with ivermectin, diethylcarbamazine and albendazole for the acceleration of lymphatic filariasis elimination in Kenya: Programmatic implementation and results of the first impact assessment
Source: PLoS Negl Trop Dis. 2024 Jul 8;18(7):e0011942. doi: 10.1371/journal.pntd.0011942 (PMC11257386; doi:10.1371/journal.pntd.0011942)
Supplement: S2 Table — (DOCX) [file pntd.0011942.s002.docx]

**S2 Table.** **Sample size determination during the impact assessment conducted in 2021.**

| **Survey Area** | **Estimated Population** | **Sample size for random cluster survey of children aged 5-9 years** | **Sample size for random cluster survey of adults 18+ years old** | **Sample size for purposively selected sites of children aged 5-9 years** | **Sample size for purposively selected sites of adults 18+ years old** |
| --- | --- | --- | --- | --- | --- |
| Lamu | 144,000 | 1380 (average of 46 kids per site) | 3150 (an average of 105 adults per site) | 50 per site (in up to 5 sites*) | 100 per site (in up to 5 sites*) |
| Jomvu | 140,000 | 1380 (average of 46 kids per site) | 3150 (an average of 105 adults per site) | 50 per site (in up to 9 sites*) | 100 per site (in up to 9 sites*) |

*The exact number of clusters that were purposively selected was dependent on the number of high-risk villages; any ‘high risk’ villages not included in the random sample formed the purposively selected clusters.
